# Thermocells for Hybrid Photovoltaic/Thermal Systems

**DOI:** 10.3390/molecules25081928

**Published:** 2020-04-21

**Authors:** Gilyong Shin, Jei Gyeong Jeon, Ju Hyeon Kim, Ju Hwan Lee, Hyeong Jun Kim, Junho Lee, Kyung Mook Kang, Tae June Kang

**Affiliations:** Department of Mechanical Engineering, Inha University, Incheon 08826, Korea; mysky24sky@gmail.com (G.S.); newjg91@nate.com (J.G.J.); juhyun4280@gmail.com (J.H.K.); juhwanlee3260@gmail.com (J.H.L.); hyeongjun2531@gmail.com (H.J.K.); lmy2415@gmail.com (J.L.); athrun93@naver.com (K.M.K.)

**Keywords:** thermocell, ferric/ferrous cyanide, carbon nanotube, photovoltaic, thermal management

## Abstract

The photovoltaic conversion efficiency of solar cells is highly temperature dependent and decreases with increasing temperature. Therefore, the thermal management of solar cells is crucial for the efficient utilization of solar energy. We fabricate a hybrid photovoltaic/thermocell (PV/T) module by integrating a thermocell directly into the back of a solar panel and explore the feasibility of the module for its practical implementation. The proposed PV/T hybrid not only performs the cooling of the solar cells but also produces an additional power output by converting the heat stored in the solar cell into useful electric energy through the thermocell. Under illumination with an air mass of 1.5 G, the conversion efficiency of the solar cell can improve from 13.2% to 15% by cooling the solar cell from 61 °C to 34 °C and simultaneously obtaining an additional power of 3.53 μW/cm^2^ from the thermocell. The advantages of the PV/T module presented in this work, such as the additional power generation from the thermocell as well as the simultaneous cooling of the solar cells and its convenient installation, can lead to the module’s importance in practical and large-scale deployment.

## 1. Introduction

There has been much research on the development of eco-friendly renewable energy technology to face and mitigate environmental pollution caused by the use of fossil fuel and to solve the problem of unstable oil prices [1,2]. Among these technologies, photovoltaic cells (PVs) with waste heat recovery to achieve effective utilization of solar energy are recognized as core technologies of the renewable energy industry, and their commercialization is underway around the world [3,4]. Due to high photoelectric efficiencies and the existence of well-established semiconductor technologies, silicon solar cells are leading the renewable solar energy technology market [5,6,7]. Efforts on exploring new materials such as non-toxic abundant perovskites as an emerging class of unconventional semiconductors have also begun to be made due to the development of more efficient and cost-effective solar cells [8,9,10].

However, the output power generated by a solar cell highly depends on its surrounding environment, which affects the operating conditions of the cell [11]. In particular, solar cells are essentially made of materials with excellent light absorption ability; thus, the temperature of the cells significantly rises during operation [12,13]. The photovoltaic conversion efficiency of solar cells is temperature dependent and decreases with increasing temperature due to the decrease in the mobility of carriers and their diffusion lengths [14]. Therefore, the thermal management of solar cells along with the exploration of new materials for photovoltaic conversion are important technical issues that must be considered to efficiently utilize solar energy.

Several potential solutions that address the need for solar cell thermal management have been proposed [15,16,17,18,19]. These solutions involve the integration of PV cells with thermal management modules, the combination of which is known as photovoltaic thermal (PVT) systems. These systems use air cooling or water cooling to cool the solar cells and increase their electrical output. For example, a water spray application over PV panel surfaces [17] and a fin-based passive cooling technique [18] were investigated as practical demonstrations for solar cell cooling. Furthermore, the advantages of integrated PVT systems over conventional solar energy plants that consist of both PV modules and solar collectors were also investigated in depth not only from a thermodynamic energy perspective [19] but also an economic perspective with regards to the installation and operation of these systems.

In addition to the research aimed at improving the efficiency and lifetime of solar cells utilizing various cooling methods, photovoltaic–thermoelectric (PV/TE) systems that can convert the heat energy absorbed by the solar cells directly into electrical energy have been a major subject of research in recent years [20,21]. To realize the full potential of obtaining additional energy using these PV/TE hybrid systems, an optimization method was proposed that is applicable to hybrid systems using different solar cells operating at ambient temperature and different solar TE generator designs [22]. Various TE devices that convert the waste heat from solar cells into electrical energy have also been extensively studied in the past decades with concrete results of actual effective means of this type of conversion.

However, the rarity of TE raw materials and the corresponding high unit cost of electric power have hampered the practical and large-scale deployment of these devices [23]. The small Seebeck coefficient typical of these TE materials (several tens to hundreds of μV/K) [24] limits the generation of practically usable voltage from the TE devices because of the small temperature difference that typically prevails between a PV panel and its surrounding. As an alternative to conventional TE devices for thermal energy harvesting, the thermocell (also known as a thermal electrochemical cell) has received much attention in recent years owing to its simple deployability in large-scale systems [25,26,27,28,29]. The high-temperature coefficient of the electrode potential on the order of mV/K renders thermocell technologies attractive for use in waste thermal energy recovery [30,31,32,33]. Other advantages of a thermocell include its ability to be used in continuous power generation, having simple components, requiring low maintenance, and being cost-effective [26,34].

In this work, a novel photovoltaic/thermal system integrated with thermocells (termed photovoltaic/thermocell (PV/T) hybrid) is proposed to serve as a proof-of-principle for a single device that can harvest solar energy from solar light and solar cell waste heat simultaneously. We fabricate a thermocell consisting of an electrolyte with a ferric/ferrous redox couple (Fe(CN)_6_^3−^/Fe(CN)_6_^4−^) and single-walled carbon nanotube (SWNT) electrodes and then integrate it directly into the back of a commercial solar panel array. The proposed PV/T hybrid not only performs the cooling function that any PVT performs but also produces an additional power output by converting the thermal energy absorbed by the solar panel into useful electrical energy through the thermocell. We also explore the feasibility of the module for its practical implementation.

## 2. Results and Discussion

### 2.1. Fabrication and Operation of the Hybrid PV/T Module

**Figure 1:**
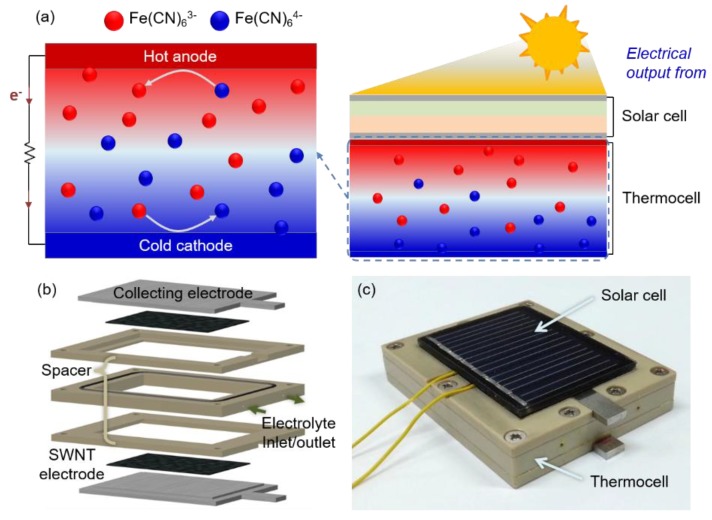
Fabrication and operation of the hybrid photovoltaic/thermocell (PV/T) module; (**a**) Schematic of the hybrid PV/T module and the thermocell operation; (**b**) Cell components and their assembly in a thermocell; (**c**) Optical image of the fabricated PV/T module.

Figure 1a shows the schematic and operation of a hybrid PV/T module. A temperature difference between the electrodes of the thermocell induces an electrical potential difference between the two electrodes where the redox species are oxidized at the anode and, through convective and thermal diffusion, reduced back at the cathode [35]. More specifically, the Fe(CN)_6_^4−^ is oxidized (i.e., Fe(CN)_6_^4−^ → Fe(CN)_6_^3−^ + e^−^) at the hot anode, which is heated due to its contact with the back of the solar cell panel, and the electrons are transferred to the electrode. The electrons produce electrical power through an external load and are transferred to the cold cathode by the potential difference between the electrodes at both ends [36]. In the cathode, which is at a relatively low temperature, a reduction reaction of Fe(CN)_6_^3−^ and the electrons occurs (i.e., Fe(CN)_6_^3−^ + e^−^ → Fe(CN)_6_^4−^), closing the circuit loop and maintaining the electrical neutrality of the electrolyte. With every mole of the reduced species that is oxidized at the hot anode, there is exactly one mole of oxidized species reduced at the cold cathode, preserving the composition of the electrolyte and allowing the cell to operate in a self-regenerative way.

Figure 1b shows the components of the thermocell presented in this work. The thermocell consisted of 1.5 mm-thick stainless steel current collectors and SWNT electrodes with an area of 1.0 cm^2^ at both ends. A 5 mm-thick layer of polyetheretherketone (PEEK) between two current collectors was used as a spacer. To prevent leakage of the electrolyte, silicone O-rings were inserted into the cell, and the entire cell was screwed together. The fabricated thermocell was then attached to the back of a commercial solar cell panel using thermal adhesive tapes. An optical image of the fabricated PV/T module is shown in Figure 1c. More details regarding the preparation of the thermocell are provided in the Materials and Methods section.

### 2.2. Evaluation of Temperature Dependence of Solar Cell Efficiency

**Figure 2:**
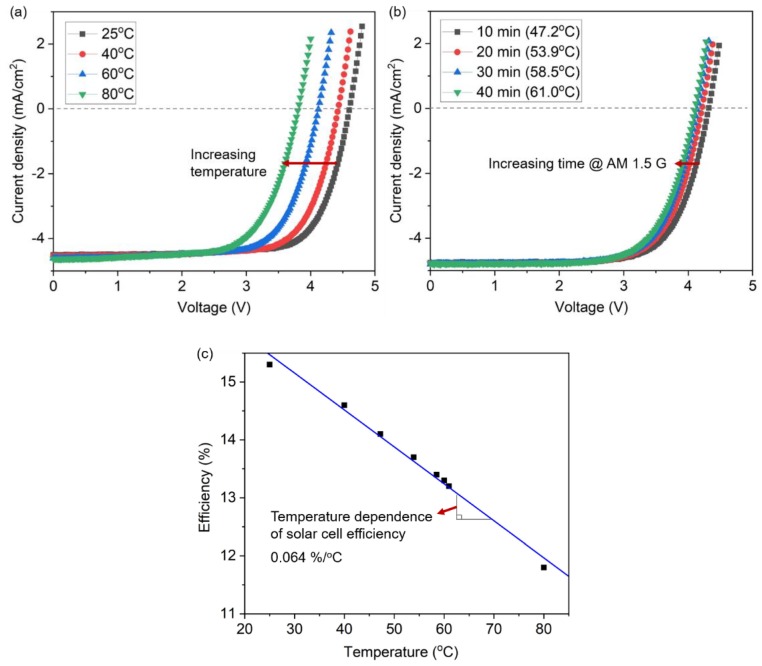
Evaluation of the temperature dependence of the solar cell efficiency: (**a**) J-V curves of the solar cell as a function of temperature; (**b**) time dependence of the J-V curves under AM 1.5 G illumination; (**c**) temperature dependence of the solar cell efficiency.

To compare the performance between a thermocell and solar cell in the PV/T module, the temperature dependence of the conversion efficiency of the solar cell was first evaluated by measuring the current density (J) and voltage (V) curves of the solar cell as a function of temperature. As shown in Figure 2a, as the temperature of the solar cell increased from a room temperature of 25 °C to 80 °C, the J-V curve shifts gradually to the left with the decrease in the open-circuit voltage (V_oc_) from 4.61 V to 3.81 V, while the absolute magnitude of the short-circuit current (J_sc_) is kept relatively constant (there is a slight increase from 4.47 mA/cm^2^ to 4.62 mA/cm^2^). The fill factor (FF) and power efficiency (η) of the solar cell also decrease correspondingly from 72.4% to 67.2% and 14.6% to 11.8%, respectively (see Table 1). The diode parameters of the solar cells, such as the reverse saturation current density, ideality factor, series resistance, and shunt resistance affect this temperature dependence of the solar cell efficiency [37].

To demonstrate a more realistic condition, a temperature increase of the solar cell was also measured with time under the standard illumination of air mass (AM) 1.5 G and a power density of 100 mW/cm^2^. The corresponding changes in the solar cell performance were investigated, as shown in Figure 2b. The temperature of the solar cell subjected to an ambient temperature of 25 °C increased gradually and reached a steady-state temperature of 61 °C after approximately 40 min of illumination. As the illumination time increased, the J-V curve shifted to the left with a decrease in V_oc_ from 4.61 V at 25 °C to 4.10 V at 61 °C. Accordingly, the FF and η of the solar cell also decreased from 73.5% to 70.4% and from 15.3% to 13.2%, respectively (see Table 2).

The experimental data for Figure 2a,b were collected and plotted in Figure 2c. From the slope of the temperature-efficiency curve, the temperature dependence of the solar cell efficiency can be determined as 0.064%/°C, suggesting that thermal management must be considered for the efficient operation of the solar cells.

### 2.3. Evaluation of Thermocell Performance

As shown in Figure 1a, the thermocell in this study consists of simple components that include an electrolyte containing a Fe(CN)_6_^3−^/Fe(CN)_6_^4−^ redox mediator and SWNT electrodes at both ends, in which the electrochemical reaction of the redox mediator occurs driven by the temperature difference between the two electrodes. The oxidation and reduction potential of the redox mediator is highly sensitive to the temperature at the reacting electrode because the free energy is a function of temperature [26]. The free energy difference (Δ*G*_rxn_) before and after the electrochemical reactions produces an electrochemical potential difference, although the reaction is reversible at standard conditions; otherwise, the potential difference is zero when the two electrodes are at the same temperature due to the reversibility of the reaction. The potential difference (Δ*V*) between two electrodes subjected to a temperature difference (Δ*T*) can be expressed by Equation (1):

(1) $$\Delta V=-\frac{\Delta G_{rxn}}{nF}=-\frac{\Delta T\cdot \Delta S_{rxn}}{nF}$$

where $\Delta S_{rxn}$ is the entropy change of the cell reaction, n is the number of electrons transferred in the reaction, and F is the Faraday constant.

From the relationship expressed in Equation (1), the temperature coefficient of the electrode potential (*S*_e_) that relates the output voltage from the cell to a temperature difference applied to the electrodes in the cell can be defined as Equation (2):

(2) $$S_{e}=\frac{\Delta V}{\Delta T}=-\frac{\Delta S_{rxn}}{nF}$$

where the magnitude and sign of $S_{e}$ are determined by the $\Delta S_{rxn}$ for a given redox reaction [38,39].

One of the reasons for choosing Fe(CN)_6_^3−^/Fe(CN)_6_^4−^ as the thermocell redox couple is that it provides a relatively high entropy change (~137 J/mol·K) corresponding to a high *S*_e_ of ~1.42 mV/K, which is almost one order of magnitude higher than the Seebeck coefficient of thermoelectric materials [40]. Another key property of the redox couple is the large exchange current density associated with the electrodes, allowing high currents to be drawn from the thermocell. Both the output voltage from the thermocell, which is related to the product of *S*_e_ and Δ*T*, and the output current determine the generated power from the thermocell. The most important advantage of the SWNT electrodes used here is the characteristic high internal surface area, which can increase the number of available reaction sites per unit of external area, resulting in an increased power density [41]. The ability to generate a high current from the thermocell also depends on the electrode performance associated with the electrochemical overpotentials for the reaction [34].

**Figure 3:**
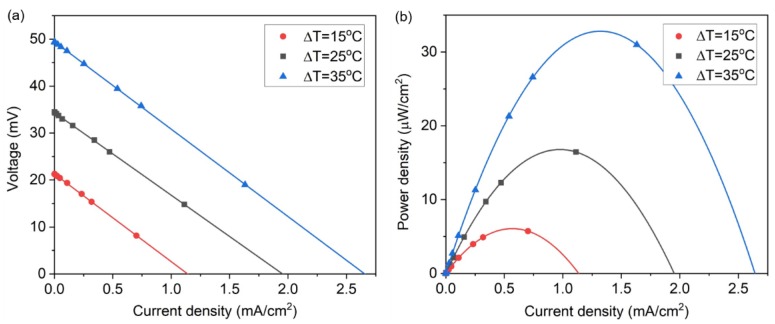
Measurement of thermocell performance according to the temperature difference at the electrodes: (**a**) J-V curves of the thermocell; (**b**) power density produced by the thermocell.

We evaluated the performance of the thermocell, as shown in Figure 3a, by measuring the J-V curves of the thermocell as a function of the temperature difference between the two electrodes (Δ*T*). In this figure, as the temperature difference increases, the V_oc_ linearly increases according to the inter-electrode temperature difference (21.3 V for Δ*T* = 15 °C, 35.5 V for Δ*T* = 25 °C, and 49.7 V for Δ*T* = 35 °C). This linear proportionality in the V_oc_ increase with respect to Δ*T* is also expected from Equation (1). The J_sc_ also linearly increases as Δ*T* increases, with the internal resistance (i.e., the slope of the J-V curve) of the thermocell maintained at a near-constant of ~18.3 ohms. The power density from the thermocell was calculated from the J-V curves and plotted in Figure 3b. The thermocell produced a maximum power density of 6.07 μW/cm^2^ for Δ*T* = 15 °C, 17.3 μW/cm^2^ for Δ*T* = 25 °C, and 33.6 μW/cm^2^ for Δ*T* = 35 °C. The performance metric of these results in terms of the temperature squared normalized specific power density is ~27.4 nW/cm^2^·K^2^.

### 2.4. Performance Evaluation of the Hybrid PV/T Module

**Figure 4:**
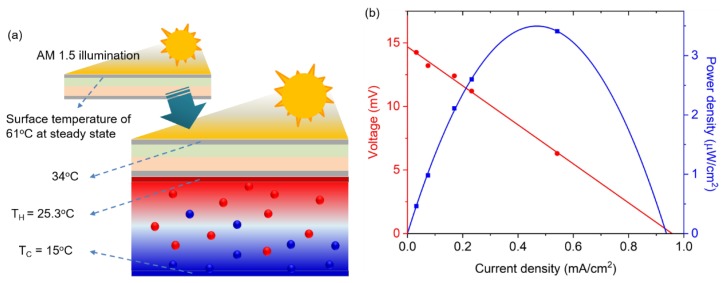
Feasibility test for the hybrid PV/T module: (**a**) temperature profile of the PV/T module under AM 1.5 G illumination; (**b**) J-V curve and the power generated by the thermocell.

Based on the temperature dependence of both the solar cell and thermocell performances, the feasibility of the PV/T module was explored under the standard illumination of AM 1.5 G, as shown in Figure 4a. The temperature at the bottom part of the thermocell was maintained at 15 °C, whereas the top part received the thermal energy from the solar cell that was heated by solar illumination, increasing its temperature. This temperature gradient is the driving force for the heat transfer that removes heat from the solar cell to the thermocell, and simultaneously, the temperature gradient in the thermocell enables the production of additional power. The ambient temperature of the PV/T module was maintained at 25 °C. Under this experimental condition, measurements of the steady-state temperature of the solar cell demonstrated a reduction from 61 °C to 34 °C, indicating that the conversion efficiency of 13.2% at *T* = 61 °C can be improved to a photoelectric conversion efficiency of ~15% at 34 °C, as indicated from Figure 2c and Table 1. With the temperature difference measured in the experiment (i.e., Δ*T* = 10.3 °C), the thermocell can generate a V_oc_ of 14.7 mV and a current density of 0.96 mA/cm^2^, producing a maximum power density of 3.53 μW/cm^2^, as shown in Figure 4b.

The measured current density from the thermocell is similar to that of the solar cell in mA/cm^2^. However, it should be noted that the output voltage of the thermocell is much lower than that of the solar cell. Specifically, the corresponding output power density of the thermocell (3.53 μW/cm^2^ for Δ*T* = 10.3 °C) is quite small compared with the average output of commercial silicon solar cells, which is roughly four orders of magnitude higher (~200–400 W/m^2^). Although this mismatch in power generation between the solar cell and thermocell can be understood by considering the varying availability of the sun’s solar and thermal energy as well as the different conversion efficiencies of the cells, it is unlikely that the output power from the thermocell will ever match that from the solar cell for maximum power transfer from both the solar cell and thermocell connected in series.

However, in the commercialization of these PV/T systems, an important requirement is to decrease the design and manufacturing costs of these systems, and this is in addition to improving the conversion efficiency of thermocells. Although thermoelectric devices have been long investigated for the direct conversion of thermal to electrical energy with many exciting advances having been made [42], device performance relative to cost has limited its application in waste heat recovery [23]. Integrating thermocell technology into PV/T systems can offer major advantages that can help mitigate these cost issues, as suggested by the comparisons of Wh/dollar of solar cells and thermocells [26,43]. Therefore, we believe that the cost-effectiveness and simple deployability of thermocells, such as the one presented in this paper, can lead to their importance in the practical and large-scale implementation of PV/T systems because remarkable advances can be made in thermocell performance.

Although this study uses a small-sized cell, thermocell performance for large-area implementation is straightforward because the output current delivered by a thermocell increases as the electrode area increases, which can be achieved by increasing the number of available reaction sites. In fact, it has been demonstrated that there is a linear relationship between thermocell performance and electrode area at a given temperature difference [30,34]. However, determining an effective thermal conductivity field [11] in the air surrounding the PV/T panel with respect to the size could be part of future research because the thermocell potential is proportional to the temperature difference with a given temperature coefficient of the redox couple. Additionally, further studies to reduce the interfacial thermal resistance between the surface of the PV panel and the thermocell using an appropriate thermal interface material may help to render the proposed PV/T system more attractive for practical implementation.

## 3. Materials and Methods

A polycrystalline solar cell panel with an area of 4 cm × 4 cm (100 mA/5 V mini solar panel) was purchased from Han Science, South Korea. A 0.4 M concentration of potassium ferricyanide (K_3_Fe(CN)_6_, Sigma Aldrich, St. Louis, MO, USA) and potassium ferrocyanide (K_4_Fe(CN)_6_·3H_2_O, Sigma Aldrich, St. Louis, MO, USA) electrolyte solution was used for the thermocell electrolyte for all experiments. Note that the concentrations provided here are total molar concentrations. The electrolyte was prepared using deionized water from a high purity deionization system (Ultra 370, YOUNG LIN Instruments, Anyang, Korea) and was degassed before use using bath sonication. To avoid the effects of electrolyte degradation, the freshly prepared electrolyte was immediately utilized for all measurements.

SWNT powders (ASP-100F, Hanwha Nanotech, Seoul, Korea), without any further purification processes, were used to fabricate thermocell electrodes in the form of sheets via a filtration process. This involved vacuum filtering an SWNT suspension in anhydrous *N*,*N*-dimethylformamide (*N*,*N*-DMF, Sigma Aldrich, St. Louis, MO, USA) onto a PTFE membrane filter (Millipore, Billerica, MA, USA, 0.2 μm pore size, 47 mm diameter), washing with deionized (DI) water and methanol, drying in vacuum, and removing the formed sheet from the filter.

The J-V curves of the solar cell were measured under an AM 1.5 G one-sun illumination (100 mW/cm^2^) using a solar simulator (Oriel Sol 3A Class) equipped with a 450 W Xenon lamp (Newport 6279NS) and a Keithley 2400 source meter. To evaluate the thermocell, the cell was placed between two fluid-heated copper plates that were connected to hot and cold thermostatic baths (A&D, AD-RC08) to provide ±0.1 °C control of plate temperatures. To establish the cooling device for the PV/T hybrid system, a copper plate was attached to the cold-side of the thermocell, maintaining a temperature of 15 °C. The J-V curves of the thermocell were measured using a computer-controlled voltage–current meter (CS310, Corrtest Instruments, Wuhan, China) with a 10 μV potential resolution and 10 pA current sensitivity from −10 to 10 V.

## 4. Conclusions

We fabricated a hybrid photovoltaic/thermocell module by integrating the thermocell directly into the back of a solar panel and explored the feasibility of the module for practical implementation. The proposed PV/T hybrid not only performs the cooling of the solar cell that current PV/Ts perform but also produces an additional power output by converting thermal energy into useful electric energy through the thermocell. Under the standard illumination of AM 1.5 G, the conversion efficiency of the solar cell improved from 13.2% to 15% by cooling the solar cell from 61 °C to 34 °C, simultaneously harvesting an additional power of 3.53 μW/cm^2^ from the thermocell. This output power density is quite small compared with the output power density of commercial silicon solar cells. Therefore, efforts to develop inexpensive but highly efficient electrodes and high-performance electrolytes should be made to render thermocell technologies commercially attractive considering that the Carnot relative efficiency should be 2% to 5% for the commercial viability of these systems [25,26,34]. In addition to these efforts to address the material requirements of a thermocell device, studies on thermocell arrays with series and/or parallel interconnections should be conducted to provide the required voltage and current for practical purposes. It is anticipated that when fully developed, the PV/T system presented in this paper can be immediately implemented at sites where existing solar panel arrays are in operation, in addition to being coupled with new module fabrication. The advent of this new approach for the thermal management of solar cells can induce significant advances in the solar energy market, bringing great advantages of cost-effectiveness and simple deployability.

## Figures and Tables

**Table 1:** Temperature dependence of the solar cell performance.

Temperature (°C)	J_sc_ (mA/cm^2^)	V_oc_ (V)	FF (%)	Efficiency (%)
25	−4.47	4.61	73.5	15.3
40	−4.54	4.43	72.4	14.6
60	−4.60	4.12	70.1	13.3
80	−4.62	3.81	67.2	11.8

**Table 2:** Time dependence of the solar cell performance under air mass (AM) 1.5 G illumination.

Time (min)/Temperature (°C)	J_sc_ (mA/cm^2^)	Voc (V)	FF (%)	Efficiency (%)
10/47.2	−4.75	4.32	72	14.1
20/53.9	−4.77	4.22	71	13.7
30/58.5	−4.80	4.14	70.1	13.4
40/61.0	−4.78	4.10	70.4	13.2
